# Abstract Mindsets Increase Believability of Spatially Distant Online Messages

**DOI:** 10.3389/fpsyg.2016.01056

**Published:** 2016-07-13

**Authors:** Hande Sungur, Tilo Hartmann, Guido M. van Koningsbruggen

**Affiliations:** Department of Communication Science, Vrije Universiteit AmsterdamAmsterdam, Netherlands

**Keywords:** construal level theory, psychological distance, online news, believability, online credibility, heuristics

## Abstract

Growing evidence from online credibility research reveals that online users rely on heuristic processes to evaluate the credibility of online information. The current paper, which is based on the construal level theory (CLT), proposes that congruency between the psychological distance of a stimulus and the way it is mentally construed can act as a heuristic for believability. According to CLT, psychologically close (e.g., spatially, temporally, socially) stimuli are represented concretely whereas psychologically distant stimuli are represented abstractly. The level of mental construals and the psychological distance of information have been shown to influence people’s truth judgments in oﬄine contexts. This study tests whether congruency between the construal level of people’s mindsets (abstract vs. concrete) and the psychological distance implied in an online message (far vs. close) enhances message believability. By partially confirming CLT predictions, we found that believability of an online news item about a distant location increased when people maintained an abstract mindset rather than a concrete one. The effect of a concrete mindset on believability was not significant for the close psychological distance condition. Our findings provide initial evidence that congruency between the construal level of people’s mindsets and psychological distance cues in online messages can act as a heuristic for believability. We discuss the potential of applying the CLT framework to the growing literature on online cognitive heuristics in the area of online information credibility.

## Introduction

People increasingly rely on the Internet as an information source for a wide range of topics that are relevant to their lives, such as health, news, commerce, and social relationships (Purcell and Rainie, 2014). Although the Internet can provide access to high-quality information, it also contains a large amount of information with questionable veracity and quality (Eysenbach et al., 2002; Kata, 2010). While factors such as the lack of quality standards and the openness to manipulation increase the amount of inaccurate information on the Internet, the lack of mechanisms for filtering and correcting information places responsibility on the user to effectively judge information accuracy (Metzger et al., 2003; Danielson, 2006; Flanagin and Metzger, 2008). Given the negative outcomes of making inaccurate veracity judgments (Brush et al., 2004; Mendoza et al., 2010; Zubiaga and Ji, 2014), it is highly relevant to understand the factors that lead people to believe the information encountered on the Internet.

Growing evidence from online credibility research revealed that people tend to process online information in quick, intuitive and effortless ways by resorting to cognitive heuristic strategies (Flanagin and Metzger, 2007; Sundar et al., 2007; Hilligoss and Rieh, 2008; Sundar, 2008; Metzger et al., 2010; Metzger and Flanagin, 2013). Instead of checking important factors such as the recentness of the information or sponsors of the information source, users prefer to rely on readily available cues, such as the visual design of a website or the number of likes (i.e., endorsement cues) to judge online information credibility (Wathen and Burkell, 2002; Fogg et al., 2003; Metzger et al., 2003; Fox, 2006). Thus far, important heuristics that stem from the technological features of media, source characteristics, and interactions among users and users’ expectations, have been proposed as factors influencing online credibility (Flanagin and Metzger, 2000; Flanagin and Metzger, 2007; Hilligoss and Rieh, 2008; Sundar, 2008; Metzger et al., 2010; Metzger and Flanagin, 2013).

In the credibility literature, especially in the media credibility literature, there has been a lack of consensus regarding the definition and measurement of credibility (Danielson, 2006; Flanagin and Metzger, 2008; Appelman and Sundar, 2016). Different components and related concepts such as trustworthiness, expertise, believability, trust, reputation, authority, quality, completeness, and accuracy have been investigated in relation to credibility. These different conceptualizations usually stem from the different foci of the researcher. Depending on the aspect of the media that is under investigation (i.e., the source, the medium or the message content), researchers prefer different operationalizations (e.g., expertise for source credibility, usability for medium credibility, and believability for message credibility) (Wathen and Burkell, 2002; Danielson, 2006). In the current study, we are specifically interested in the perception of message credibility that is independent of source- and medium-related credibility perceptions. Therefore, we examine believability, a core component of message credibility (Appelman and Sundar, 2016) that is considered to be the overarching and consistent aspect of credibility (Tseng and Fogg, 1999; Flanagin and Metzger, 2000, 2008; Wathen and Burkell, 2002). Given the extensiveness of believability, we expect that the heuristics that influence believability will similarly effect the other closely related concepts used in relation to message credibility.

The present paper extends the online credibility research by proposing the construal level theory (CLT) as a framework that allows the systematic investigation of a novel set of heuristics on which users may rely to assess the believability of online messages (Liberman and Trope, 1998; Trope and Liberman, 2003, 2010). CLT is a psychological theory that explains the relationship between the distance of events from people’s immediate reality (psychological distance) and the way they are represented in people’s minds (construal level) (Soderberg et al., 2015). The relationship between the construal level and psychological distance has been shown to influence people’s truth judgments in oﬄine contexts (Bar-Anan et al., 2006; Hansen and Wänke, 2010; Wright et al., 2012). Importantly, a large amount of online content readily conveys psychological distance cues about spatial locations, time, people and likelihoods as well as cues that initiates concrete or abstract construals. Following the assertions of CLT and building on the online user’s tendency to rely on heuristic cues, the current study tests whether the congruency between people’s mindset construals and the psychological distance cues in online messages can act as a heuristic that influences the believability of online messages.

CLT examines the psychological processes that allow people to move beyond their immediate reality of *here, now, self, certainty*, and enable them to consider distant places, the future or the past, other people and hypothetical situations (Trope and Liberman, 2010). According to CLT, people can transcend their reality by forming abstract mental representations, or construals regarding distant events that are psychologically distant (Liberman and Trope, 2008). CLT explains the relationship between mental construals and the psychological distance of events (or objects or people) and aims to predict the way in which this relationship can influence evaluations, perceptions, and behaviors of people. Thus far, CLT has been successfully applied to explain various cognitive and behavioral outcomes in areas such as visual perception, memory, categorization, probability estimates, marketing, consumer behavior, and creativity (Trope and Liberman, 2010; Soderberg et al., 2015) and is gaining recognition among communication scholars as well (Nan, 2007; Lutchyn and Yzer, 2011; Katz and Byrne, 2013; Ellithorpe et al., 2015).

Construal level refers to how concretely or abstractly a stimulus (e.g., event, object, person, or situation) is represented. A low-level construal is a concrete, contextualized representation that involves several specific subordinate exemplars. A high-level construal on the other hand, involves abstract, decontextualized, and superordinate representations (Liberman and Trope, 2008). For instance, the act of “being environmentally friendly” can be represented by several concrete actions such as “conserving water”, “biking instead of using the car” or “recycling” (low-level construal). It can also be represented abstractly as a goal, such as “making the world a better place” (high-level construal). While low-level construals entail information on *how* to do something, high-level construals relate to the question of *why* to do them (Eyal et al., 2009). Therefore, low-level construals involve exemplars that apply to specific contexts (e.g., conserving water, recycling) while high-level construals preserve the essential meaning that would apply in different situations (e.g., making the world a better place).

The second main concept, psychological distance, refers to the subjective distance of events, objects or actions from people’s immediate reality (Liberman et al., 2007b). People’s experiences of the world are generally centered in the *here, now*, the *self*, and in *reality*. According to the CLT, things that are removed from this center of experience spatially, temporally, socially or hypothetically, are psychologically distant (Trope and Liberman, 2010). These four dimensions are interrelated as they share a common meaning based on psychological distance.

According to CLT, the concepts of psychological distance and construal level are also interrelated. While people can think about psychologically close events concretely and in detail, psychologically distant events need to be represented with more general features and abstractly, as people lack detailed knowledge (Liberman and Trope, 1998). For instance, thoughts about meeting with a friend that will take place *tomorrow* will likely involve concrete, low-level details such as a location, the hour of the meeting, or the activity in which you will be engaging. However, thoughts of meeting with a friend that will take place *next year* will more likely involve more general and abstract representations such as “having a good time” (Liberman et al., 2002). Thus, abstraction preserves the essential features of events and allow people to contemplate beyond their immediate experience of which they have less information (Liberman and Trope, 2008). This relationship between the construal level and psychological distance is bidirectional whereby higher levels of construal are shown to bring more psychologically distant events to mind (Liberman et al., 2007a). For instance, thinking about “going out with friends” will more likely bring close people and places to mind, while a higher-level representation of the same event, such as “having fun,” can involve various additional activities involving long distances.

The interrelatedness between the construal level and psychological distance has been demonstrated in various empirical studies (Liberman and Trope, 1998; Bar-Anan et al., 2006; Fujita et al., 2006a; Wakslak et al., 2006; Herzog et al., 2007; Liberman et al., 2007a; Wakslak and Trope, 2009). Bar-Anan et al. (2006), in a series of implicit association tasks, showed a preference for congruent matching between construal level and psychological distance. They found that participants responded faster when concrete words were matched with words signifying close psychological distances and when abstract words were matched with words denoting far psychological distances. Other studies showed that psychologically distant events and situations are described more abstractly, categorized more broadly and perceptually abstracted more compared to psychologically close events (Liberman and Trope, 1998; Fujita et al., 2006a; Wakslak et al., 2006). Similarly, people are found to base their predictions on far psychological distances more on abstract information (Henderson et al., 2006; Nussbaum et al., 2006).

Construal level theory has been linked to the concept of believability with a few studies that measured subjective truth estimates in oﬄine contexts (Hansen and Wänke, 2010; Wright et al., 2012). One of the main findings of these studies is that low-level construals enhance truth perceptions. People primed to have low-level construal mindsets evaluated marketing statements as truer compared to people primed to have high-level construal mindsets (Wright et al., 2012). Similarly, Hansen and Wänke (2010) demonstrated that concrete information is typically seen as more truthful. Across three studies, they showed that concretely versus abstractly written statements (Study 1 and 2) and low-level versus high-level mindsets lead to higher truth ratings (Hansen and Wänke, 2010).

Another main finding of these studies was that the congruency of elements within the CLT framework enhanced perceptions of believability. Congruency within the CLT framework was created in several ways. First, congruency between participants’ construal level of mindset and linguistic construal of target statements (i.e., reading abstractly written statements while maintaining an abstract mindset) has been shown to enhance the believability of statements (Henderson et al., 2006). Second, congruency of different psychological distance dimensions presented in statements has been shown to enhance believability (Wright et al., 2012). This congruency was created by presenting proximally congruent psychological distance information such as close temporal and close social distance (“yesterday” and “ you”) or far temporal and far social distance (“last year” and “your friend”) in statements. People perceived such statements to be truer compared to statements involving proximally incongruent psychological distance information. Finally, congruency was created by matching the linguistic construal level and perceptual psychological distance (Hansen and Wänke, 2010). Concretely or abstractly written statements were presented with spatially close or distant locations of pictures on a computer screen. The participants gave higher truth ratings to statements when the spatial distance matched the construal level of the sentences (i.e., when a concretely written statement appeared in close spatial distance).

The underlying reason for these enhanced truth perceptions is suggested to be the ease of processing or processing fluency that is experienced during the congruent matching of psychological distance and construal level (Reber and Schwarz, 1999; Alter and Oppenheimer, 2006; Hansen and Wänke, 2010; Wright et al., 2012; but also see: Kim et al., 2009 for a boundary condition). Processing fluency refers to the metacognitive ease or difficulty experienced during information processing (Alter and Oppenheimer, 2008; Oppenheimer, 2008; Reber and Unkelbach, 2010), and it is considered to be an important metacognitive cue that guides judgments. Previous research has shown that experiencing fluency can lead to positive feelings such as confidence and liking as well as higher truth judgments (Reber and Schwarz, 1999; Winkielman et al., 2003; Alter and Oppenheimer, 2009).

Given that online users typically rely on available cues to judge the veracity of information encountered online (Fogg et al., 2003; Flanagin and Metzger, 2007; Hilligoss and Rieh, 2008; Metzger et al., 2010) and the fact that online content may commonly include psychological distance cues (e.g., locations, time, social actors, and likelihoods) as well as cues for concreteness and abstraction, we tested whether the CLT framework can be used to identify new heuristics for online believability. While the link between CLT and subjective truth estimates has been established in oﬄine contexts (Hansen and Wänke, 2010; Wright et al., 2012), the application of CLT to online believability has been limited. Thus far, to our knowledge, only one study applied CLT to the processing of tweets and suggested that online users find tweets that depict proximally similar psychological distance dimensions more likely and less surprising compared to tweets involving incongruent psychological distance dimensions (Sungur et al., 2015). However, the effects of construal level and psychological distance congruency on online believability have not been investigated thus far. Therefore, the current study sets out to investigate this core CLT premise in relation to online believability.

While the relationship between construal level and psychological distance has been demonstrated in oﬄine contexts (Liberman and Trope, 1998; Bar-Anan et al., 2006; Fujita et al., 2006a; Wakslak et al., 2006; Herzog et al., 2007; Liberman et al., 2007a; Wakslak and Trope, 2009), it is also important to examine this relationship in an online context. First of all, the typical relationship between psychological distance dimensions and construal level can be different on the Internet because the implications of distance differs between oﬄine and online environments (Guadagno et al., 2013). For instance, spatial distance may not always carry the same implications in online and oﬄine settings (Kaltenbrunner et al., 2012). While a far spatial distance limits direct experience in the physical world, spatially distant stimuli can be experienced in a similar fashion as spatially proximate stimuli on the Internet (e.g., in a video chat). As the association between construal level and psychological distance becomes stronger and more generalized with repeated experiences (Trope and Liberman, 2010), these atypical experiences on the Internet may influence this relationship. Secondly, online environments contain unique psychological distance cues that does not exist anywhere else. For example, user profiles on social network sites contain social distance cues (e.g., number of common friends or connections), as well as temporal and spatial distance cues (e.g., time stamps, check-in to locations). Online communication and online content are abound with such psychological distance cues (e.g., user generated content, geolocations, online reviews). The unique processes and consequences of making credibility judgments on the Internet (Metzger et al., 2003; Danielson, 2006; Flanagin and Metzger, 2008), the potentially different working mechanism of CLT assertions and the unique psychological distance cues provided by the online technologies give merit to the testing of CLT in online contexts.

In the present study, we tested the effect of the congruency between the construal level of the mindset and the psychological distance information in online messages on message believability. We hypothesized that when the construal level of the participants’ mindset is congruent with the psychological distance information in the message, the believability of the message increases. Specifically, we hypothesized that a message involving high psychological distances would be perceived as more believable when participants are in a high-level (abstract) construal mindset rather than in a low-level (concrete) construal mindset. Similarly, we hypothesized that a message involving close psychological distance information would be perceived as more believable when participants are in a low-level (concrete) construal mindset than a high-level, (abstract) construal mindset.

## Materials and Methods

### Participants and Procedure

One hundred seventy-nine participants completed the experiment via an online survey listed on Amazon’s Mechanical Turk in return for $0.40. Prior to the data collection, the inclusion criteria were set to identify the eligible data for analysis. According to these criteria (see the parentheses for the number of participants that did not fulfill each criterion), the participants were required to reside in the US (*n* = 1), correctly respond to a question designed to measure attention to the task (i.e., “Please select option 2 on this question to show that you are paying attention”) (*n* = 6), have spoken English for a minimum of 6 years (*n* = 0), not take a break during or after the construal level manipulation task (*n* = 4), follow the instructions of the construal level manipulation task adequately (*n* = 15), and do not have extreme reading durations for the target news item containing the psychological distance manipulation (*n* = 11) (1 participant did not fulfill multiple criteria). The final sample consisted of 143 participants (96 women, 47 men; *M*_age_ = 38.7 years, and *SD*_age_ = 14.2 years).

We employed a 2 (construal level: low vs. high) × 2 (psychological distance: close vs. far) between-subject factorial design. The participants were first randomly assigned to a low or high construal level mindset manipulation condition. Subsequently, for an ostensibly unrelated task, the participants randomly received the close or far psychological distance manipulation embedded within an online news item. Afterward, the participants received the believability measure. On the next page psychological distance perceptions toward the news report was measured. Finally, after other relevant questions the participants were debriefed on the aim of the study. The experiment required approximately 10 min to complete.

### Materials

#### Construal Level Manipulation

According to the action identification theory (Vallacher and Wegner, 1987), actions involve hierarchies because they can be represented in terms of higher or lower level goals. Each action (e.g., locking the door) has a superordinate, abstract-level representation that relates to the higher-level goal and answers to the question of why to engage in that action (e.g., security). Actions also have subordinate, concrete-level representations that describe how to perform that action (e.g., turn the key in a lock). According to CLT, thinking of *how* to do an action primes low-level representations whereas thinking of *why* to do an action leads to abstract and high-level representations (Wakslak and Trope, 2009; Trope and Liberman, 2010). The construal level was manipulated by an online version of the How and Why task, which is a procedure designed and validated by Freitas et al. (2004). The How and Why task has been used to place people into low- or high-construal level mindsets by prompting them to think about *how* or *why* to perform certain actions (Freitas et al., 2004; Fujita et al., 2006b; Wakslak and Trope, 2009; Wright et al., 2012). Following the typical How/Why procedure, the participants received a goal (“Improve and maintain physical health”) and were asked either *how* or *why* they would like to achieve that goal. The participants had to answer to *how* or *why* four times, each time responding to a previously self-given response. In the *how* condition, the goal was provided at the top of the page with arrows pointing downward with the *how?* prompts. Successively answering the *how* questions requires participants to think increasingly concretely and focus on low-level, subordinate activities relating to the goal (e.g., Improve and maintain physical health, *how?* →Go to gym, *how?* →By getting a gym membership, *how?* →By searching for gyms on the Internet…). In the *why* condition, the goal was provided at the bottom of the page, with arrows pointing upward with the *why?* prompts. Providing successive answers to the *why* questions requires increasingly abstract thinking, in which one must consider the high-level, superordinate goals (e.g., Improve and maintain physical health, *why*? → To live longer, *why?* → To spend time with my family, *why?* → To be happy…).

The manipulation check for the how/why task was carried out in a similar fashion to the procedure used by Fujita et al. (2006b). Two coders (the first author and a second coder who was unaware of the aim of the study and blind to the conditions), coded the responses given during the construal level manipulation task (the second coder coded a random subsample consisting of 30% of participants’ responses). A response received a score of +1, if it fulfilled the criterion “X by Y”, X being the response and Y being the prompt (e.g., live longer by maintaining and improving physical health). A response received a score of -1 if it fulfilled the criterion “Y by X” (e.g., maintain and improve physical health by going to the gym). A response received a score of 0 if it did not follow these two rules. At the end, a final score was calculated by summing the 4 scores. The interrater reliability between the two coders was very high: Kappa = 0.87 (*p* < 0.001), 95% CI (0.80, 0.94). Overall, the two groups’ scores significantly differed from each other, *t*(141.89) = 61.32, *p* < 0.001, Cohen’s *d* = 9.69, (How condition: *M* = -3.63, *SD* = 0.91 and Why condition: *M* = 3.77, *SD* = 0.59). As previously mentioned, based on this manipulation check, fifteen participants (How condition: *n* = 2 with a score of 0, *n* = 3 with a score of -1, *n* = 3 with a score of -2 and Why condition: *n* = 6 with a score of 2) were removed from the analysis for failing to adequately follow the instructions.

#### Psychological Distance Manipulation

**FIGURE 1:**
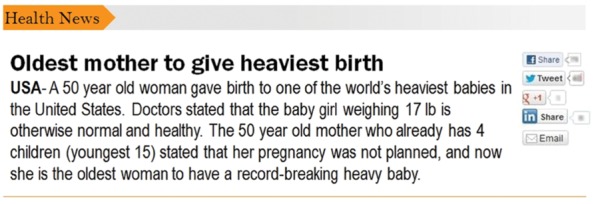
**Spatially close news report.** The psychological distance was manipulated by varying the spatial location of the event in a news report. The spatially close condition depicted an event taking place in the US because the participants also resided in the US. The spatially distant condition occured in Suriname. Aside from the names of the two countries, the news reports were identical.

Psychological distance was manipulated by varying the spatial distance depicted in a news report. The report was a short story presented as a screenshot of a news item from the health news category about an older woman giving birth to a heavy baby (**Figure 1**). The news was fictional (participants were unaware of this); however, it was selected as a possible event that could happen anywhere in the world. The event either took place in the USA (spatially close condition) or Suriname (spatially distant condition). The news reports were otherwise identical. The source of the news item was described as an online news blog without specifying further details.

The topic of the news item and the countries were selected based on a previous study. In an online study, we asked US residents (*N* = 47) how spatially close or distant they felt toward several countries (1 = very near, 7 = very far) including the US itself. Suriname was one of the countries that was rated spatially distant (*M* = 6.06, *SD* = 1.32), and this was significantly different from the distance the participants felt toward the US [*M* = 1.2, *SD* = 0.97, *t*(46) = 21.9, *p* < 0.001]. We also presented participants with news topics without specifying where they occurred and asked how believable they found the content of the news stories in general (1 = not at all, 7 = very much). The news item used in this study received consistent mid-scale point believability scores, with approximately 60% of all scores receiving ratings of 3, 4, or 5 which suggests that, in general, people did not think this story could be absolutely true or false (*M* = 3.62, *SD* = 1.62).

### Measures

Based on previous studies (Hansen and Wänke, 2010; Wright et al., 2012), believability was assessed by a single-item that asked participants to rate how believable they found the news item (1 = unbelievable, 7 = believable). We also asked the participants about the spatial, temporal and social distance they felt toward the described event (“The event described in the news report felt like it is happening: close to where I am/current time/to people like me”; 1 = strongly disagree, 7 = strongly agree). Furthermore, we measured the participants’ mood, the degree to which they trust online news in general, and how often they read online news. Finally, we asked the participants to indicate what they thought the study was about to check for awareness of our hypotheses.

### Ethics Statement

All procedures were approved by the Department of Communication Science of the Vrije Universiteit Amsterdam, and the study adheres to APA ethical guidelines (American Psychological Association Ethics Committee, 2010). All data were collected anonymously and analyzed aggregately. The participants joined the experiment voluntarily via a link published on Amazon’s MTurk in return for a monetary reward. MTurk provides information regarding the topic, requirements, length and potentially offensive content of the listed studies, and participants are free to choose the study they wish to participate in. We informed the participants that they would be required to perform a short thinking exercise followed by a survey on their evaluations of a news report. The participants were ensured that the data would be collected and analyzed anonymously and that their responses would not be traced back to them. The participants were allowed to withdraw from the study anytime they liked without any consequences. At the end of the study, the participants were debriefed and were told that the news report they evaluated was actually altered. They were encouraged to reach out to the authors in the event of any questions or if they are interested in knowing the outcome of the study.

## Results

The participants who read about the event taking place in the US perceived the event as happening spatially closer to them (*M* = 2.26, *SD* = 1.22) than participants reading about the event in Suriname (*M* = 1.83, *SD* = 1.28), *t*(141) = 2.06, *p* = 0.04, Cohen’s *d* = 0.34. The temporal distance and social distance did not differ between the two spatial distance conditions (USA: *M*_temporal_ = 4.34, *SD*_temporal_ = 1.81, Suriname: *M*_temporal_ = 4.37, *SD*_temporal_ = 1.83, *t*(141) = 0.09, *p* = 0.92, Cohen’s *d* = 0.02, and USA: *M*_social_ = 2.18, *SD*_social_ = 1.46, Suriname: *M*_social_ = 1.91, *SD*_social_ = 1.43, *t*(141) = 1.09, *p* = 0.28, Cohen’s *d* = 0.19).

**FIGURE 2:**
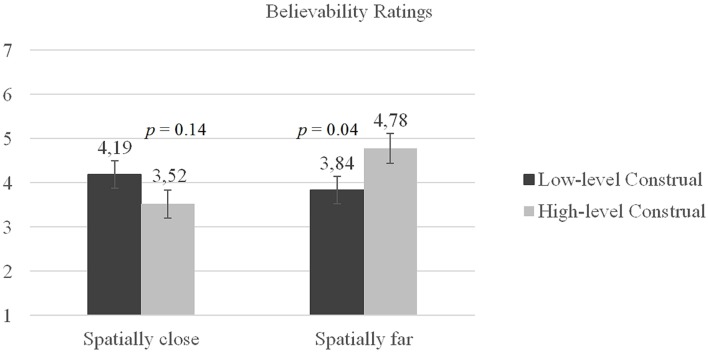
**Believability ratings.** Believability ratings for the news report. An abstract, high-level construal mindset increased the believability of the news report regarding a spatially distant country significantly more than a concrete, low-level construal mindset. The construal of the mindset did not have a significant effect on the believability of the news report about the spatially close country.

The effect of the construal level and psychological distance on the believability of the online news was analyzed with a two-way ANOVA. The results revealed no main effects of construal level, *F*(1,139) = 0.18, *p* = 0.67, $\eta_{p}^{2}$ = 0.001, and psychological distance, *F*(1,139) = 1.97, *p* = 0.16, $\eta_{p}^{2}$ = 0.014. However, as hypothesized, we observed a significant interaction between the construal level and the psychological distance on the believability score, *F*(1,139) = 6.15, *p* = 0.01, $\eta_{p}^{2}$ = 0.04 (**Figure 2**). Simple main effect analyses revealed that the participants found the news report about Suriname (the spatially distant condition) significantly more believable when they were in a high-level construal mindset (*M* = 4.78, *SD* = 1.93) rather than a low-level construal mindset (*M* = 3.84, *SD* = 1.88), *p* = 0.04, Cohen’s *d* = 0.49. For the US news report (the spatially close condition), the participants reported higher believability when they were in a low-level construal mindset (*M* = 4.19, *SD* = 2.01) rather than a high-level construal mindset (*M* = 3.52, *SD* = 1.88), but this difference was not significant, *p* = 0.14, Cohen’s *d* = 0.34.

## Discussion

This study showed some support for our hypothesis that congruency between the users’ construal level and psychological distance information within online messages enhances the believability of online messages. Specifically, the participants in a high-level, abstract mindset believed the online message implying far psychological distance more than the participants in a low-level, concrete mindset. Additionally, although not statistically significant, the participants in a low-level, concrete mindset reported higher believability for the online message implying close psychological distance than participants in a high-level, abstract mindset. The observed pattern of means and the significant interaction between construal level and psychological distance on the believability of the online news message are consistent with the predictions of CLT (Trope and Liberman, 2010).

While we observed the expected effect for the far spatial distance news item, the predicted effect was not significant for the close spatial distance news item. There can be several reasons for this outcome. One of the explanations for this could be the failure to induce sufficient levels of closeness with the selected stimulus. We used participants’ country of residence (the US), which is an objectively closer spatial location compared to Suriname, to induce spatial proximity. Although, the participants reported feeling significantly spatially closer to the news item about the US compared to the news item about Suriname, the mean ratings do not show strong perceptions of spatial proximity for the US. This can be due to the wide range of locations implied when a large country such as the US is used as a spatial marker. The interaction between the low-level construal mindset and close psychological distance is essentially based on people’s concrete, detailed observations and knowledge regarding their immediate environment (Trope and Liberman, 2010). Therefore, using a rather general location may have limited the strength of the interaction in the current study. Similarly, the two news items were not perceived to be different with regard to social and temporal distances. This may not be surprising given that there was no specific cue regarding these dimensions in the news report. Nevertheless, the social distance ratings reveal that participants in the close condition felt a high level of social distance. The fact that the presented event was not rated as uniformly psychologically proximate or distant (across all dimensions) might have dampened our findings. Another reason for the current findings may relate to the difference between the reliance on heuristics by experts and novices. Previous research has shown that experts tend not to rely on construal-level heuristics as novices do (Kim et al., 2009). It is possible that some participants in the close condition would deduce that the story is not believable given that they did not hear about it. Finally, some previous research suggested that low-level construals may be less prone to the influence of psychological distance manipulations than high-level construals because they are already highly concrete and contextualized (Pennington and Roese, 2003; Nan, 2007).

The current study, is one of the first to apply CLT to explain online believability judgments and it complements the literature on heuristic approaches to online credibility (Hilligoss and Rieh, 2008; Sundar, 2008; Metzger et al., 2010; Metzger and Flanagin, 2013). While previous research identified important heuristics that stem from the technological features of media, the characteristics of the source and the interaction among users, the CLT approach can add novel heuristics to this repertoire. A recent study already showed that online users do indeed utilize available psychological distance cues and expect congruent psychological distance information within online messages (Sungur et al., 2015). Our findings extend this research by examining another type of heuristic — mindset-distance congruency — which also builds on the available cues in the content of online messages which can, in an automatic fashion, influence believability judgments.

Our findings are in general consistent with previous research on CLT and believability in oﬄine contexts that showed consistency between the mindset and the psychological distance (Hansen and Wänke, 2010; Wright et al., 2012) and extends this research by showing evidence for a mindset-psychological distance congruency effect. Our findings did not support the previous findings that low level construals always lead to higher truth judgments (Hansen and Wänke, 2010; Wright et al., 2012). One reason for this could be the limitations we mentioned regarding inducing necessary levels of psychological proximity. Another reason could be the different methodologies used in these previous studies. While Hansen and Wänke (2010) used linguistic concreteness, Wright et al. (2012) did not use any psychological distance manipulation to test the effects of construal level. Thus far, the previous research has investigated believability in a CLT framework by examining the relationship between mindset construal and linguistic construal in oﬄine messages (e.g., reading abstractly/concretely written messages with an abstract/concrete mindset) or the relationship between psychological distance and linguistic construal (e.g., presenting abstractly/concretely written statements in distant/close locations on a computer screen) (Hansen and Wänke, 2010; Wright et al., 2012). Therefore, the current study also contributes to the literature on CLT in demonstrating the effect of congruency between people’s mindset construal and psychological distance with regard to the believability of messages. Furthermore, our findings showed that even though the Internet removes many geographical boundaries, spatial distance is still a relevant factor that can influence perceptions in online communication.

The current findings also provide support for the potential of applying CLT to the area of persuasive communication. In their CLT of mobile persuasion framework, Katz and Byrne (2013) proposed several methods of applying CLT assertions to mobile technology to deliver persuasive messages. For instance, they argued that when the construal level of the mindset and the psychological distance in a message are congruent — or, as they refer to it, when they are *cue congruent* — then the persuasiveness of the message should increase. Mobile devices can both track the location of users and calculate their distance from areas of interest (e.g., distance from a cigarette vendor for a person trying to quit smoking), measure or shift the construal level of people and deliver a customized message. More specifically, according to the mobile persuasion framework, the GPS function might signal to an intervention application that a user with a dieting goal is near a fast food restaurant. The application might then assume that the user is having a concrete/low-level representation of the food-choice because the user is at close spatial distance. As a result, the application could display a persuasive health message that matches the user’s presumed construal level of the mindset. Alternatively, existing mindset manipulations might be adapted and embedded within mobile application games that can be used to alter a recipient’s mindset (Katz and Byrne, 2013) before displaying a matching persuasive message. We believe that our findings also provide preliminary empirical insight into these theoretical propositions by Katz and Byrne (2013).

### Limitations and Future Research

The current study manipulated the construal level of the mindset with a topic that was independent of the content of the target message (i.e., thinking of how or why to maintain physical health). Although the prior research has shown that this is an effective method and that the construal level of the mindset can influence unrelated decisions (Fujita et al., 2006b; Wakslak and Trope, 2009), a more natural and direct way of manipulating the construal level may be better suited for applications outside of experimental situations. The alternative would be to create a mindset and psychological distance congruency within the same message. In situations where the topic of the online message allows (e.g., climate change, dieting), it may be possible to pose questions about why or how to accomplish a related goal and cue psychological distance within the same message. For instance, in their study White et al. (2011), primed the construal level by highlighting the “ways” (i.e., low-level construal) or “reasons” (i.e., high-level construal) that recycling behavior can be changed. Building on this approach, one can embed psychologically close cues with a low-level construal priming (i.e., ways to recycle today) and psychologically distant cues with the high-level construal priming (i.e., reasons to recycle this year). Another direct way of matching construal level and psychological distance can be by shifting construal level linguistically or by using words to represent high psychological distance stimuli and pictures for low psychological distance stimuli (Amit et al., 2009). Future research should explore these alternatives strategies to increase the applicability of influencing online believability judgments via construal level and psychological distance congruency. Another limitation of the current study is that we tested our hypothesis in a single-message context. The extent to which our findings can be generalized to other messages thus remains to be seen. Future research could profit from testing this hypothesis by using different (multiple) messages (also see Reeves et al., 2015; Slater et al., 2015). Finally, future studies exploring the potential underlying mechanism of processing fluency would improve our understanding of the CLT mechanisms.

## Conclusion

The present study showed that congruency between users’ mindset construal and spatial distance information within online messages can enhance believability judgments. Given the tendency of users to rely on available cues to judge the veracity of encountered online information, this study introduced CLT as a novel framework that can expand the existing research on the heuristic factors that influence the believability of online messages.

## Author Contributions

HS Conceived and designed the experiments, collected the data, analyzed the data, contributed reagents/materials/analysis tools, and wrote the manuscript. TH Conceived and designed the experiments, contributed reagents/materials/analysis tools, and wrote the manuscript. GK Conceived and designed the experiments, contributed reagents/materials/analysis tools, and wrote the manuscript.

## Conflict of Interest Statement

The authors declare that the research was conducted in the absence of any commercial or financial relationships that could be construed as a potential conflict of interest.
